# A Historical Legacy for Universal Health Coverage in the Republic of Korea: Moving Towards Health Coverage and Financial Protection in Uganda: Comment on "Health Coverage and Financial Protection in Uganda: A Political Economy Perspective"

**DOI:** 10.34172/ijhpm.2023.7434

**Published:** 2023-02-13

**Authors:** Chun-Bae Kim

**Affiliations:** ^1^Department of Preventive Medicine, Yonsei University Wonju College of Medicine, Wonju, South Korea.; ^2^Hongcheon County Hypertension and Diabetes Registration and Education Center, Hongcheon, South Korea.

**Keywords:** Universal Coverage, Financial Burden, Health Insurance, Benefit Package, South Korea

## Abstract

Since 2001, when Uganda abolished user fees to improve the accessibility of healthcare, out-of-pocket costs still account for 42% of total health expenditure. Even if universal health coverage (UHC) is achieved on the demand-side, government authorities face political and economic challenges due to soaring burden of diseases. Therefore, this study aimed to re-analyze the implementation process according to three pillars by World Health Organization (WHO) based on Korean UHC-related articles. In terms of breadth, the national health insurance (NHI) in Korea UHC was established from 1977 for employees to 1989 for self-employed. In terms of depth, benefit packages in Korea UHC have expanded from essential medical services to expensive care (ultrasono, computerized tomography, etc) including benefit period. Finally, in terms of height of coverage, the government has tried to relieve financial burden of households with catastrophes and enhance benefit plan for major diseases till now. This historical legacy for UHC in Korea can pose lessons to policy-makers in developing countries including Uganda and Ghana.

## Introduction

 In 2022, which marks the 44th anniversary of declaration of Alma-Ata by the World Health Organization (WHO), our international community is paying new attention to universal health coverage (UHC), which allows citizens of all countries to receive good quality medical services, including appropriate responses to the coronavirus disease 2019 (COVID-19) pandemic. Unlike European industrialized countries, which already began social security systems based on tax or social health insurance in the late 19th century, a valid case of UHC in the world would bring about important implications for low- and middle-income countries (LMICs). In 2001, the Uganda government launched the “free healthcare” policy by abolishing user-fees in public facilities, thus improving access to health services for the poor. In spite of this policy, out-of-pocket expenses still account for 42% of total health expenditure and financial catastrophes have not declined over the last two decades. Thus, the insurance sector is under-developed, contributing little to health financing for Ugandan people. Nannini et al^1^ have reviewed Uganda’s negotiation process for health financing reform through a qualitative study method with a political economy focus. However, this effort was limited in broadening its understanding due to the lack of comprehensive access to three essential technical ways (breadth, depth, and height) for universal coverage expansion identified in the WHO’s 2008 report^2^ through its assessment of primary healthcare in LMICs for the past 30 years.

 Korea, which overcame colonial rule and became independent after World War II, was among the poorest countries due to the Korean War. Accordingly, Korea, which has been granted official development assistance including the health sector for more than 40 years since the 1950s^3^, has been converted to a member of the Organisation for Economic Co-operation and Development (OECD) today due to economic development. In addition, Korea achieved ambitious universal coverage in 12 years, the fastest in the late 1980s during the economic leap forward. This story is considered to be the cornerstone for establishing politically sustainable strategies to achieve UHC in LMICs and to be practical in Uganda’s political and economic implementation process. Therefore, this qualitative review intended to reconstruct the development process according to the three technical pillars described by the WHO based on information of the National Health Insurance Service (NHIS)^4^ and Korean UHC-related articles.^5-11^

## Basic Concept of Universal Health Coverage

**Figure 1:**
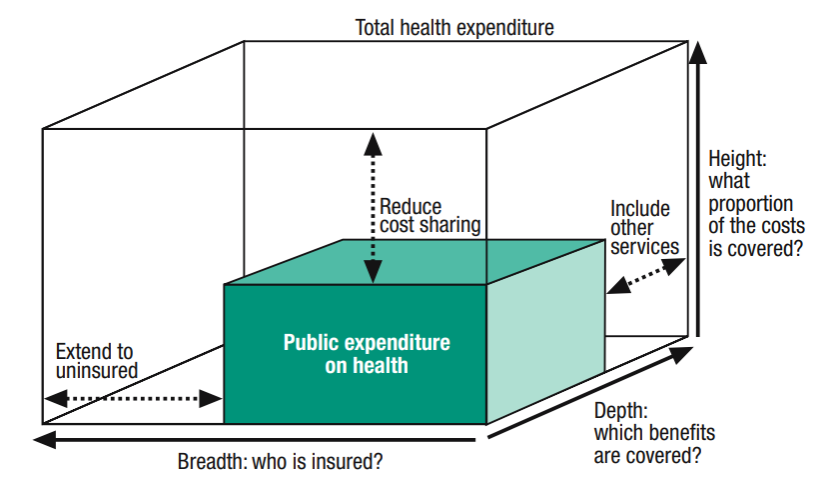
Three Ways of Moving Towards Universal Coverage.Source: World Health Organization.^2^

 Since 2001, the implementation of health coverage and financial protection in Uganda has raised various political economic issues^1^ which might be of interest to other countries (including Ghana) in Africa. The move towards UHC is to expand coverage in three technical ways^2^ described by the WHO as shown in Figure 1. The operational definitions of breadth, depth, and height of coverage should be considered in the process of expanding UHC. The breadth of coverage – the proportion of the population that enjoys social health protection – must expand progressively to encompass the uninsured, ie, the population that lacks access to services and/or social protection against financial consequences of taking up healthcare. The depth of coverage must also grow, expanding the range of essential services that are necessary to address people’s health needs effectively, taking demand and expectations as well as resources that the society is willing and able to allocate to health into account. The height of coverage is the portion of health-care costs covered through pooling. Pre-payment mechanisms must also rise, diminishing reliance on out-of-pocket co-payments at the point of service delivery.

## Moving Towards Universal Health Coverage in Korea

###  1. Who Is Insured? 

 Korea did not have initiatives to develop a healthcare system fully under successive political turmoil and social instability such as Japanese colonization and American military occupation before and after World War II. In particular, in the 1950s, when the Korean War had no health infrastructure at all, South Korea received intensive aid from US Foreign Operations Administration, including United Nations Korean Reconstruction Agency. Through such support, some health facilities such as health centers and public clinics were created and health services were provided in a full patient-burden manner. A medical education system was also established due to the Minnesota project.^3^

 In 1963, the Korean military government enacted the Medical Insurance Act and introduced a voluntary health insurance system based on government subsidies in terms of breadth of coverage. However, at that time, per capita income was less than $200. Thus, no one could directly purchase voluntary insurance. By 1965, only two manufacturing companies (Korea Integrated Chemical, Bongmyeong Graphite Mining Industry) voluntarily offered employer based health insurance. Voluntary regional medical insurance was also implemented in a few areas (included Jeonju, Geoje-, Wanju-, Chunseong-, and Ganghwa-county) that had implemented pilot projects for primary healthcare, which began as part of the international non-governmental organization’s official development assistance support since 1965.^3^ Overall, the health insurance was not actually implemented until the mid-1970s due to the nation’s weak economic and social infrastructure.

 In 1976, the Medical Insurance Law was amended substantially in order to include mandatory enrolment of the insured and dependents in Korea.^5,6^ From the beginning, the medical insurance system adopted a family-based membership, with dependents becoming members of the scheme when their household head as the insured was enrolled in. The medical insurance for employees was based on workplaces and that for self-employed was based on their region of residence. When the 4th 5-Year Economic Development Plan (1976-1981) was promulgated, social development was emphasized and two initiatives, the Medical Aid Program and compulsory health insurance for employees at large firms, were started. It was economically easy to pay insurance premiums. Corporations with more than 500 employees were the first target to be covered by medical insurance in 1977. At that time, gross domestic product (GDP) per capita was US$ 1052.9. A Medical Aid Program was started for the poor in 1977. Government employees and teachers joined the medical insurance program in 1979. The workplace-based insurance was extended to firms with more than 300 employees in 1979. It was further extended to firms with more than 100 employees in 1981, to those with more than 16 employees in 1983, and to those with more than 5 employees in 1988.

**Figure 2:**
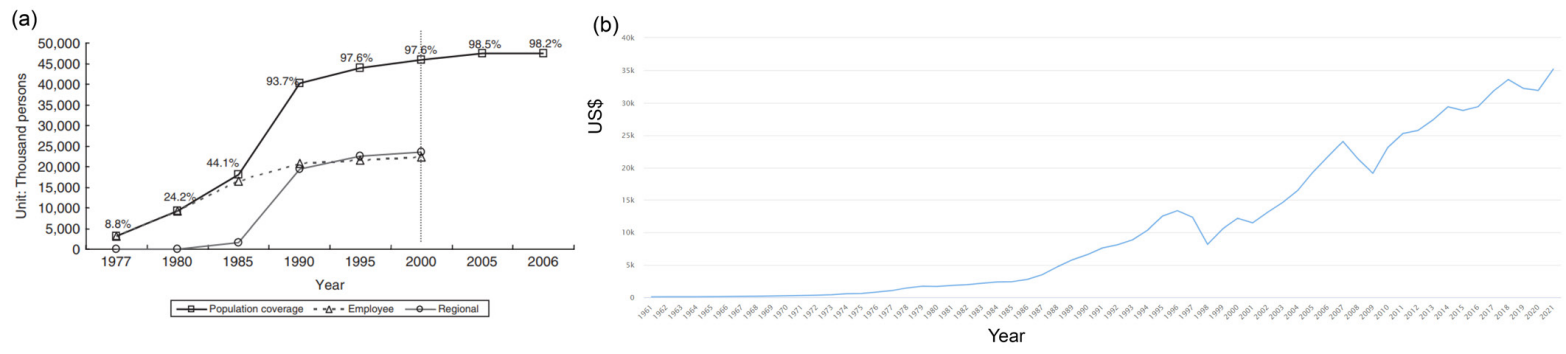
Population Coverage Trend of Universal Health Insurance (a) and Gross National Income Trend (b) in South Korea. Source: (a) Kwon.5^5^ (b) Gross national income per capita according to National Accounts in Bank of Korea.

 In 1978, Korea formally endorsed the primary healthcare strategy as the WHO’s goal of ‘Health for All’ by the year 2000.^3,7^ To extend medical insurance to self-employed, the government implemented a pilot program in three rural areas (Gunwi-, Hongcheon-, and Okgu-county) in 1981 among more than 300 target districts. It then implemented the pilot program in three additional areas (Ganghwa- and Boeun-county, Mokpo-city) in 1982. The compulsory medical insurance program achieved universal coverage of the population by including rural self-employed in January 1988 and urban self-employed in 1989 (Figure 2). In 1989 after Seoul Olympic when universal coverage was achieved nationwide, per capita income was US$ 5800.6. In this way, all Koreans at demand-side were covered by the national health insurance (NHI) for the first time in 12 years in the perspective of coverage’s breadth under strong government leadership and governance. According to the distribution of insurance coverage among Koreans in 1990, it consisted of 37.4% of workers, 9.5% of the poorest and vulnerable class, 10.1% of public officials and (school) teachers, 14.5% of rural residents, and 28.5% of urban self-employed people.

 In 2000, major structural reforms were made to the NHI system. A total of 370 insurance societies were merged into one organization, the National Health Insurance Corporation. This integration brought together a single insurer for government employees, teachers, and their dependents, 142 insurers for employees and their dependents, and 227 insurers for self-employed.^6^ Since 2004, standards for applying insurance benefits have been expanded or eased to active-duty soldiers, inmates, and foreigners’ dependents. Almost 100% of the people are currently receiving UHC now.

###  2. Which Benefits Are Covered? 

 In terms of depth of coverage, health insurance policies were also made to expand benefits for essential medical services (including maternal and child health, family planning, communicable diseases control, etc) offered. Naturally, when insurance began, all public health facilities (including public health centers, health sub-centers, and health posts) in the public sector and all medical institutions, including clinics, hospitals, and general hospitals in the private sector, were compulsory designated as care facilities in 1979. The non-transparency of the Ministry of Health and Society and the passive role played by the legislature under the Yushin dictatorship enabled this compulsory designation system in which doctors’ rights were violated^8^ at supply-side.

 Based on results of the pilot program for oriental medicine insurance in 1984, benefits of oriental medicine services were fully expanded in 1987. At that time, South Korea underwent a political revolution in the era of democracy in the wake of the pro-democracy movement. In 1989, prescription drugs dispensed at a pharmacy were added to the benefit package. The insurance benefit period also extended the number of days allowed for treatment, from 180 days per year in 1994 to an additional 30 days for several years. Then all restrictions were finally removed in 2000. In addition, the separation system between prescribing and dispensing of drugs was fully introduced in 2000 after a pilot program in 1984. Accordingly, doctors were prohibited from dispensing drugs. They were limited to prescribing drugs, leaving drug dispensing to pharmacists.

 A long-term care insurance (LTCI) was created in 2008 to provide coverage for facility- and home-based long-term care for older people who could pass the eligibility test based on functional and cognitive disability.^9^ It is a separate fund from the NHI, although it is managed by the same agency (NHIS). The contribution to LTCI is a fixed percentage of NHI contribution (currently 8.5% of NHI). It covers 9% of those aged 65+. Since 2016, LTCI has been in deficit due to a rapid increase of eligible older people.^10^

 The policy for strengthening health insurance coverage was announced in 2017 to make a reliable country where nobody needs to worry about medical expenses. Currently, the use of expensive medical equipment (computerized tomography, magnetic resonance imaging, ultrasound, etc), prevention and rehabilitation services for diseases and injuries, home-visit care by doctors, and health checkups for regional residents aged 20 or older are also provided as insurance benefits. After the WHO declared COVID-19 pandemic in 2020, the NHIS also covers diagnosis costs with the health insurance fund to prevent suspected patients from avoiding or rejecting testing due to financial burden. The health insurance covers 80% of treatment expenses for confirmed COVID-19 cases due to the power of UHC.^11^ The central government covers the remaining 20% (Table). Thus, any Korean can receive treatment without any financial burden^4^ in terms of depth of coverage. The benefit package has expanded. Public share of financing has also continuously increased.

**Table:** Expansion of Universal Health Coverage According to Three Technical Pillars in South Korea

**Breadth of Coverage**		**Depth of Coverage**			**Height of Coverage**			
**Year**	**Health Insurance Policy**	**GNI (US$)**	**Year**	**Health Insurance Policy**	**GNI (US$)**	**Year**	**Health Insurance Policy**	**GNI (US$)**
1965-1966	Two firms established voluntary insurance societies for employees	109.6-131.0	1979	Forced designation of medical care institutions	1720.1	1977	Announcement for establishing medical insurance prices and drug prices	1052.9
1965-1976	A few counties (Jeonju, Geoje, Wanju, Ganghwa, Chunseong, etc) implemented a voluntary regional insurance program	109.6-830.4	1984	Pilot program of separation of medical and pharmaceutical practice (Mokpo-city) Pilot program for oriental medicine insurance (Cheongju-city, Cheongwon-county) Change benefits period to 180 days per year/(same disease)	2378.9	1985	Copayment system as cost-sharing to outpatient-based medical care below clinic	2426.5
1977	^*^Mandated to provide health insurance for industrial workers *Firms with >500 employees*	1052.9	1987	Nationwide expansion of oriental medicine insurance	3512.2	2004	Out-of-pocket payment with copayment ceiling	16 476.9
	^**^Medical aid program^a^ mandated: free care for the poorest and reduced charges for the poor		1989	Medical benefits for pharmacies	5800.6	2005	To reduce high medical costs for cancer, heart disease, cerebrovascular disease, and rare incurable diseases	19 261.8
1979	*Firms with >300 employees (mandatory)*	1720.1	1994	Expand benefits period from 180 days to 210 days per year for the insured aged ≥65 years and dependents Expanded delivery benefit coverage from spouse of insured to all dependents	10 357.0	2006	Expanding reduction of insurance premiums for the vulnerable class	21 663.7
	^***^Government workers and teachers/school staffs							
1981-1982	*Firms with >100 employees (mandatory)*	1857.1-1973.0	1995	Expand benefits period from 180 days to 210 days per year for all insured ones Abolition of restriction of medical benefit period such as the elderly ≥65 years, registered disabled, dependents, and person of national merit	12 522.4	2018	Supporting catastrophic medical expenditure	33 563.7
	Regional demonstration program^b^ for self-employed, farmers, workers in small business, and their dependents							
1983	*Firms with >16 employees (mandatory)* *Firms with >5-15 employees (optional)*	2174.9	1996	Expand benefits period from 210 days to 240 days per year Include as insurance benefits category (CT)	13 350.6			
1984	Expanding the range of recognition of dependents (father-in-law and mother-in-law)	2378.9	1997	Expand benefits period from 240 days to 270 days per year	12 334.2			
1987	Expanding the range of recognition of dependents (brother, sister, spouse of direct descendant)	2378.9	1998	Expand benefits period from 270 days to 300 days per year	8189.5			
1988-1989	*Firms with >5 employees (mandatory)* Regional compulsory medical insurance (mandatory) ^****^For rural residents	4717.7-5800.6	1999	Expand benefits range (prevention and rehabilitation for diseases and injuries)	10 548.8			
	^*****^For urban residents							
2004	Expanding benefits target to active-duty soldiers	16 476.9	2000	Nationwide separation of medical and pharmaceutical practice Finally eliminated all restrictions of benefit period for medical services	12 178.7			
2005	Expanding benefits target to inmates in prisons	19 261.8	2008	LTCI (≥65 years old)	21 345.3			
2011	Relaxing benefits requirements for Korean nationals residing abroad, alien dependents	25 255.8	2017	Announced the policy for strengthening health insurance coverage	31 734.1			
			2018	Establish benefits of home-visit care by doctors for the insured or dependents Expand targets of health examinations to regional insured or dependents ≥20 years old	33 563.7			
			2020	NHIS facilitates quick testing and diagnosis of suspected COVID-19 cases NHIS covers all treatment expenses for confirmed COVID-19 cases	31 880.6			

Abbreviations: COVID-19, coronavirus disease 2019; GNI, Gross national income per capita according to National Accounts in Bank of Korea; CT, Computer tomography; NHIS: National Health Insurance Service; LTCI, long-term care insurance. Composition of beneficiaries in 1990 (million, %): ^*^16.5 (37.4), ^**^4.2 (9.5), ^***^4.5 (10.1), ^****^6.4 (14.5), ^*****^12.6 (28.5). ^a^ Medical aid program: Coverage for low income, indigent, disabled and the elderly provided by the government. ^b^ Regional (demonstration) medical insurance program: (1) 1981 - In three rural areas (Gunwi-, Hongcheon-, and Okgu-county) started on a mandatory basis. (2) 1982 - Expanded to three additional areas (Ganghwa- and Boeun-county, Mokpo-city).

###  3. Another Face of Universal Health Coverage in Medical Market

 What was most remarkable about Korea’s achievement was that its ‘Health for All’ policy was rapidly achieved within a free market-based healthcare system with a predominantly fee-for-service payment.^7^ After implementing the compulsory medical insurance system in 1977, the government notified medical insurance prices for only 55% to 60% of the customary price, even if it was limited to some insurance patients. Accordingly, medical institutions in private sector have maintained a profit structure by covering losses from the rest of patients who do not have insurance or by providing services of various non-insurance benefits.^8^

###  4. What Proportion of the Cost Is Covered? 

 In terms of height of coverage, the government applied a high out-of-pocket co-payment that pays 20% and 30% of the total medical expenses to inpatients and outpatients, respectively. In order to curb the utilization of medical care due to moral hazard effect, the government raised the outpatient co-payment level from 36.2% in 1985 to 46.7% in 1986.^12^

 In order to relieve households of the financial burden due to health equity after the establishment of the democratic system, a ceiling of co-payments depending on patients’ income was implemented in 2004, a benefit enhancement plan for four major diseases (cancer, heart disease, cerebrovascular disease, and rare incurable diseases) was implemented in 2005,^13^ and insurance premiums for the vulnerable were reduced in 2006. According to results of the Korean Welfare Panel Study 2008–2016, households facing catastrophic health expenditure (CHE) were less likely to exit from poverty to near-poverty at CHE thresholds of 20%~30%.^14^ Thus, the Korean government has tried to raise the benefit coverage by supporting households with CHE since 2018 (Table). At that time, GDP per capita was US$ 33 563.7 in Korea.

## Towards Achievement of Universal Health Coverage Together

 The result of research by Nannini et al^1^ can help us identify major obstacles in the political economy perspective against the implementation of health financing reforms for UHC in Uganda. However, since it was a qualitative study that attempted a multi-level analysis based on related documents and stakeholder interviews, the health policy might have limitations in practical acceptance and application. Considering the influence of political economy on a healthcare system, reforms can allow us to disentangle country-specific experience and historical legacy related to financial protection for UHC in the world.

 South Korea has the NHI system with the experience of achieving UHC in 12 years, starting medical insurance in 1977. The background was supported by rapid economic development along with political legitimization of the authoritarian regime amid the unique temperament of Koreans. While universal insurance coverage is a reality, significant problems remain as the nation grapples with problems of ever rising demand and soaring medical costs. Current health expenditure per capita was US$ 3406 (current USD) in 2019, which was an equivalent of 8.2% of GDP in Korea. In the 2015-2019 period, average per capita spending on healthcare grew by an average of 2.7% across OECD countries. However, Korea continued to show strong average growth of 7.8%.^15^ In conclusion, this unique experience in Korea over the last seven decades illustrates several challenges which are likely to present themselves topolicy-makers in LMICs (including Uganda and Ghana) wishing to move toward a UHC system in three ways recommended by the WHO.

## Acknowledgments

 This work is currently receiving a grant (#2022-51-0372) of ‘Mid-term Evaluation of the 2nd Phase of Maternal and Child Health Promotion project in Volta and Oti Regions, Ghana’ from the Korea Foundation for International Healthcare (KOFIH).

## Ethical issues

 Not applicable.

## Competing interests

 Author declares that he has no competing interests.

## Author’s contribution

 CBK is the single author of the paper.
